# Stuck between medals and participation: an institutional theory perspective on why sport federations struggle to reach Sport-for-All goals

**DOI:** 10.1186/s12889-022-14230-5

**Published:** 2022-10-11

**Authors:** Thomas De Bock, Jeroen Scheerder, Marc Theeboom, Bram Constandt, Mathieu Marlier, Tom De Clerck, Annick Willem

**Affiliations:** 1 Department of Movement and Sports Sciences, Ghent University, Ghent, Belgium; 2 Policy in Sports & Physical Activity Research Group, KU Leuven, Leuven, Belgium; 3 Department Sport & Society, VUB, Brussels, Belgium; 4 Department of Exercise, Health and Sport, LUNEX University, Differdange, Luxembourg

**Keywords:** Sport-for-All, Sport federations, Institutional theory, Institutional logics

## Abstract

**Background:**

Sport-for-All emphasizes that every individual has the right to participate in sport. Despite all efforts to deliver Sport-for-All during the past decades, studies indicate that sport participation rates have been stagnating, whereas social inequalities in sport continue to exist. By applying an institutional theory lens, this study sheds light on how the dual mission of sport federations, i.e., providing Sport-for-All and high performance sport, affects the Sport-for-All projects of Flemish sport federations (e.g., amount of projects and target groups). In particular, Sport-for-All projects have to reduce barriers to engage in the sport system and be supported by a sport federation. Furthermore, this study seeks to better understand the impact of the underlying institutional logic on the institutional pressure and legitimacy of the sport federations.

**Method:**

This study implemented a cross-sectional field study in sport federations. In particular, the sport federations selected for our study are the 47 Flemish sport federations. Both qualitative (i.e., document analysis) and quantitative research methods (i.e., a new questionnaire was developed based on institutional theory) were applied in the study.

**Results:**

Results indicated that sport federations are important partners in support of Sport-for-All projects, but also suggested that there is a discrepancy between the projects of the high performance-oriented and the Sport-for-All-oriented federations. Specifically, the high performance-oriented federations were targeting youth participants, whereas Sport-for-all-oriented federations aimed to reach disadvantaged groups. Furthermore, the results indicated that high performance-oriented federations endured more institutional pressure than Sport-for-All-oriented federations.

**Conclusion:**

The results of our study indicated that the Sport-for-All projects of performance-oriented federations are often more superficial compared to Sport-for-All oriented federations, and that the latter federations play an important role in attaining public health targets. Moreover, policymakers should consider how they can optimize the role of the performance-oriented federations in the Sport-for-All delivery (e.g., they could function as a bridge to guide participants who prefer a less competitive setting towards Sport-for-All oriented federations).

## Introduction

The societal advantages of sport participation are widely recognized, as illustrated by outcomes such as improved social skills and public health [1]. Moreover, practicing sport has been associated with higher levels of physical activity, improved mental health [2], and higher social capital [3]. With that consideration in mind, the Sport-for-All Charter was launched in 1975. The main aim of this Charter was to provide more sporting opportunities for as many Europeans as possible. Furthermore, the Charter has triggered national governments to promote Sport-for-All among all layers of society because of the positive health aspects of sport [4]. In the democratizing process of the national sport policies, national governments relied on the national sport federations.

National sports federation and their members (i.e., sports clubs) continue to be one of the most important players in implementing Sport-for-All. In particular, sport federations are urged to assist in the delivery of Sport-for-All, by offering and supporting Sport-for-All projects [5]. Although these projects have brought new groups of participants to the federation-organized sport, several challenges have persisted over the years [6, 7]. Sport participation rates have been stagnating in recent years, as the organized sport sector struggles to reach disadvantaged groups [8]. Furthermore, most sport federations deal with an internal duality as they have to combine ‘Sport-for-All’ with ‘high performance’ sport [9, 10]. Sport-for-All is focused on lowering barriers to sport and democratizing sport participation, whereas high performance sport is attained through athlete achievement in major international elite competitions [11]. According to institutional theory, both priorities are integrated as institutional logics in federations and are therefore shaping the interests, identities, values, and assumptions of these organizations [12, 13].

In light of the challenges that are associated with balancing these institutional logics in sport federations, the following research questions are formulated: How many Sport-for-All projects are sport federations currently supporting? (RQ1); Does the underlying institutional logic of the federations (being a Sport-for-All logic or a high performance logic) have an impact on the outcomes of their Sport-for-All project (e.g., in terms of target groups these projects aim at)? (RQ2); and What implications does the underlying logic have on sport federations’ current responses to their institutional environment? (RQ3). The study was conducted in Flanders (i.e., the largest, Dutch-speaking, northern part of Belgium) and responds to the call of Skille [14] for more theory-guided and empirical research to increase our understanding of dominant logics in sport and their implications. Moreover, this study meets the recommendation of Eime et al. (2022) to collect and analyze data concerning sport participation to better serve policy evaluation and redirection of sport policies [15].

## Literature review

### The rise of the Sport-for-All

The origins of the Sport-for-All idea reside in the post-Second World War era in which sport participation was largely dominated by young, achievement-oriented white males, mostly from the middle and upper social class [16–20]. Hence, a first considerable appeal to implement a more inclusive and organized sport policy was elaborated by the Council of Europe. In 1975, the Council launched the Sport-for-All Charter, thereby taking the lead role in advocating a broader and more democratized sport participation in Europe [21, 22]. The Sport-for-All Charter soon became well-established throughout Europe, emphasizing that every individual has the right to participate in sport [6, 23]. Furthermore, the Charter enhanced the assignment that national governments of the European Union had to coordinate and promote sports among all layers of society, including disadvantaged communities [4, 24, 25].

In Europe, the Norwegian and Flemish (Belgium) governments were the first governments to practically implement the Sport-for-All idea. Although, the responsibility to deliver Sport-for-All is in many European countries shared among many actors, such as local authorities and municipalities, voluntary organizations, and sport federations and their members (i.e., the sport clubs) [5], the implementation of Sport-for-All still remains a responsibility of the sport federations [5]. More precisely, sport federations are privileged organizations in offering Sport-for-All [13] and thus federations develop Sport-for-All projects which they implement directly or via their clubs.

According to Coalter [26], sport projects that aim to improve social inclusion, which is the main aim of Sport-for-All projects, may embody several outcomes. In particular, Coalter [26] distinguishes a non-definitive list of five outcomes. A first outcome encompasses the removal of barriers to sport participation for specific target groups, as some of them still encounter exclusionary mechanisms such as discrimination, high membership fees, and financial costs [27–30]. Secondly, these projects can provide opportunities to develop sporting skills. Thirdly, the projects can provide opportunities to overcome the gap between recreational participation and competition. Fourthly, extra training and support of coaches are considered important in the projects. Coaches can fulfil a key role in motivating specific target groups to become and stay active in sport [31, 32]. A fifth outcome is the establishment of partnerships with schools, sport clubs, and the wider community. Partnerships often add value to improve sport participation of specific target groups [26, 33].

### Decline of the Sport-for-All idea?

Although the first decennia of Sport-for-All were considered fruitful and the augmented sport participation contributed to several societal and public health targets, such as improving social capital or controlling the rising obesity levels in the general population [34–36], several researchers, such as Green [37], Haudenhuyse [38], Vandermeerschen (40, 41), and Hylton and Totten [29], are critical for the contemporary Sport-for-All delivery [29, 37–40]. According these authors, Sport-for-All has been a guiding ethos for decades, but its momentum as guiding idea has been declining [37, 39, 41, 42]. Moreover, several challenges exist for the contemporary Sport-for-All delivery of sport federations. Firstly, sport federations are confronted with stagnation in sport participation rates and physical inactivity among the general population remains a major concern [4, 7, 8, 42–47]. Secondly, there is still an underrepresentation of specific target groups in sport federations [48, 49]. A first group that is underrepresented are disabled people [43]. Recent research in different European countries (i.e., Belgium, England, and Italy) has demonstrated that there is a gap between the sport participation rates of disabled people and non-disabled people [28, 49–52]. A second group that is underrepresented in organized sport are seniors [28, 34, 43]. In particular, research indicates that sport participation tends to decrease with aging [53]. Baker et al. (2010) state that given several negative stereotypes towards aging (i.e., associations between getting older and being less capable and weaker), the drop-out of seniors is not surprising. Especially, the more competitive context of sport clubs appears a less suitable sport context for this group [28, 54]. The third group that is engaging less in organized sport are people living in disadvantaged situations, such as people from lower socioeconomic status (SES) groups or people with a migration background [48]. Literature indicates that the social integration of disadvantaged communities is often challenging for the organized sport sector [55]. In challenging times like now, with a migration crisis in 2015 [56, 57] and the Russian – Ukrainian war that started in 2022, the number of disadvantaged groups further increases, and sport may act as a critical mechanism to cope with these challenges.

In general, Hylton and Totten [29] concluded that despite its potential, the goal of Sport-for-All has never been fully achieved, and successes remain incomplete and partial. Gains have been made, but massive social inequalities remain as none of the actors contributing to Sport-for-All have been able to sufficiently reach these target groups [29]. This is especially the case for sport federations because these organizations are faced with a dual-mission of delivering Sport-for-All on the one hand, and high performance sport on the other hand [58]. According to De Bosscher et al. (2015), high performance sport is highly regulated and technical, and focused on obtaining top results in major international elite competitions (i.e., Olympics) and professional leagues [11]. This contrasts with Sport-for-All which is less technical, for a broader population, with effectiveness being based on totally different criteria. Sport federations often grapple to deliver both outcomes. Moreover, encouraging this dual-mission has constituted tensions in sport federations [59, 60]. In the past, policy makers often claimed that focusing on Olympic and elite sport success would automatically trigger the general population to become more active in sport (i.e., trickle-down effect). However, Bauman et al. (2021) indicated that this potential trickle-down effect is not always emphasized in the Olympic legacy, and thus chances to create a switchover from elite sport to the general population are often not optimized [10]. Moreover, failure to deliver both -Sport-for-All and elite performance- on the level of sport federations contributes to the suboptimal delivery of Sport-for-All [58]. This study questions whether Flemish sport federations indeed struggle to reach specific target groups in their Sport-for-All projects and whether this struggle is due to having to balance a Sport-for-All and a high performance logic.

## Theoretical framework: institutional theory

To analyze the tension between Sport-for-All and high performance, institutional theory is applied as overarching theoretical framework. Several reasons justify the application of institutional theory in sport. Firstly, one of the issues that makes sport attractive to apply institutional theory is the large amount of stakeholders and ‘license-holders’ of sport [61]. Secondly, all sport federations are embedded in an institutional context and are subject to pressure from key suppliers of resources, their members, competitors, and regulatory agencies [62]. Moreover, sport federations encounter more governmental interference in comparison to many other organizational settings [5]. Finally, the framework provides us with an understanding of how federations acquire social acceptance and authorization by adopting the norms and expectations of their institutional environment [63, 64].

The fundamental concern that institutional theory aims to acknowledge is ‘why and with what consequence do organizations exhibit particular organizational arrangements that defy traditional rational explanations.’ (Greenwood et al. 2017, p. 8). To this aim, institutional theory distinguishes multiple key elements, which we will shortly describe in the following part [61, 65–67].

The first element implies that organizations are embedded in and influenced by an institutional context. An institutional context can be understood as ‘those organizations that, in the aggregate, constitute a recognized area of institutional life: key suppliers, resource and product consumers, regulatory agencies and other organizations that produce similar services or products*’* [66]. The institutional context represents an intermediate level between organizations and society. It forms the area in which field-level actors directly interact and influence one another in a structured manner [68]. According to institutional theory, the institutional context is characterized by isomorphic processes. The central idea of isomorphism is that the institutional context constrains organizations to resemble other field-level actors that face the same set of conditions and pressures them to adopt specific practices and processes [66].

Secondly, the institutional context includes divergent belief systems that are operating inside the environment, while providing the organizing principles of that environment. These principles are known as institutional logics [69–72]. Institutional logics are defined as ‘the socially constructed, historical patterns of material practices, assumptions, values, belies, and rules by which individuals produce and reproduce their material subsistence, organize time and space, and provide meaning to their social reality’ [71, 73]. According to Reay and Hinings [72], institutional logics are meaningful theoretical constructs, because they provide understanding of the connections that create a sense of common purpose and unity in the institutional context. Institutional theorists subscribe the interpretation that the institutional environments are organized to a dominant institutional logic [72–74]. According to institutional theory, institutionalized logics are taken for granted, widely accepted, and thus resistant to change [61, 74].

The third key element of institutional analysis is that by addressing the dominant institutional logics, organizations hope to receive legitimacy and ultimately to survive in their environment [75]. The struggle for legitimacy, defined here as ‘*a generalized perception or assumption that the actions of an entity are desirable, or appropriated within some socially constructed system of norms, values, beliefs, and definitions*’ (Suchman 1995, p. 574) plays a decisive role in the emergence of dominant logics and is one of the core insights of institutional theory [61, 76–78].

### Institutional theory in sport

By applying those characteristics, it becomes clear that the organization of sport is indeed a context characterized by multiple—and at times contending—logics [79–83]. The research on institutional logics can be linked to the remaining challenges of Sport-for-All. More specifically, research on the Scandinavian context contributes to explaining the Sport-for-All policies, by analyzing the dichotomous relation between different logics in sport clubs more closely.

Stenling and Fahlén (2009, 2016) stated that Swedish sport clubs are characterized by a struggle between institutional logics. They identified three dominant logics: (a) the Sport-for-All logic, (b) a result-oriented logic, and (c) a commercialization and professionalization logic. They indicated that, although the Swedish sport system argues to be mainly Sport-for-All-oriented, the sport clubs are usually an expression of the result-oriented and professionalization logic. They conclude that there is an order of logics where the Sport-for-All logic is overshadowed by the other two. One of their arguments is that rewards given for adhering to some logics are simply higher, or perhaps more easily understood, than for others. While it is easy to discover whether one won a tournament, achievements in terms of reaching Sport-for-All goals are more difficult to be materialized and therefore less visible [83]. Skille [14] elaborated on the tension between the Sport-for-All and the competitive logic. He concluded that, as long as competitiveness is the dominant focus of sport, it implies that Sport-for-All and other logics are hard to realize. Skille [14] raised the call that further research is necessary to enhance our understanding of sport logics and – not at least – their implications. This study contributes to that call and explores how sport federations deal with the dichotomies relation between the Sport-for-All and high performance logic, while also shedding light on how this relation impacts their Sport-for-All projects.

## Methodology

### Study design

The study applied a cross-sectional field study of sport federations. The outcome of the study is a snapshot of the position of Sport-for-All projects in the institutional context of sport federations.

### Sample selection

The sport federations selected for our study are the 47 Flemish sport federations subsidized by the Flemish government. To be more precise, Flanders counts 70 registered sport federations, of which 47 sport federations are subsidized by the Flemish government. The other 23 sport federations are registered, but not subsidized (e.g., bowling, ice skating, and wushu) [84]. Sport in this context is defined by Sport Flanders as a physical activity, with a cardiovascular training effect, that is executed by a person in a healthy, ethical and medical responsible climate, and organized by a sport federation [85]. Three reasons can be presented to support why only subsidized federations are taken into account. Firstly, the group of 47 subsidized sport federations focus on the most popular sports (e.g., soccer, gymnastics, and athletics). As such, they comprise the highest membership rates. Secondly, these federations are obliged to disclose their policy and operational documents on their websites and to update their website frequently, which is in contrast to the non–subsidized sport federations. Thirdly, the subsidy entails obligations, such as providing Sport-for-All and high performance sport. By only including the subsidized federations, we have a homogenous sample of federations that are facing a similar set of obligations based on the subsidies these federations receive. In the population of 47 subsidized sport federations, 40 sport federations address one specific sport. The other seven federations are the so-called multisport federations, representing several sports [86].

### Data collection

The data collection consisted of two phases. In the first phase, the focus was put on the mapping of the Sport-for-All projects, comprising an analysis of three types of data sources. Firstly, a document analysis was conducted, including all policy plans, annual reports, reports of board meetings, and reports of the regulatory agency (i.e., Sport Flanders) in order to map all Sport-for-All projects supported by the sport federations. Secondly, the websites of the sport federations were examined. These latter data sources included information about the aims of the Sport-for-All projects, how the projects were developed, and information about partnerships, and the number of participants. Thirdly, the mapping was supplemented with data from a questionnaire, in which federations were invited to list all the Sport-for-All projects they support. This triangulation method provided a complete overview of the Sport-for-All projects of sport federations in Flanders (Belgium) [87].

To select a Sport-for-All project, we applied two selection criteria. Firstly, the project has a direct affiliation with one of the Flemish subsidized sport federations. As the study’s focus is on sport federations, Sport-for-All projects supported by one of the sport clubs—but not by the federation were not included in the mapping. Secondly, the project reduces barriers for participants (e.g. distance barriers, financial barriers, and information barriers).

In addition to the mapping of projects, our study aims to indicate how the outcomes of Coalter [26] are integrated into the Sport-for-All projects. As mentioned in the literature review, Coalter distinguished a non-definitive list of outcomes perused by sport programs that try to improve social inclusion, which were: (a) to reduce barriers to sport participation, (b) the provision of opportunities to develop sporting skills, (c) the provision of a recreational competition, (d) extra support program for coaches, and (e) the establishment of partnerships with schools, sport clubs, and the wider community.

The second phase of data collection aligned with the second and third research question on how sport federations dealt with the tension balancing a Sport-for-All and high performance logic. Given the lack of validated scales measuring the key elements of institutional theory in sport, we developed a new questionnaire to provide an answer to our research questions. Four consecutive steps were taken to compile our questionnaire: (a) we started with drafting questions based on how institutional theorists described institutional pressure, dominant logic, resource allocation, and legitimacy; (b) we explored the scientific literature to find (qualitative) questionnaires which originated from institutional theory and compared these questions with our first draft version; (c) a sport panel was composed, which consisted of several researchers, (ex)-staff members of federations, and sport managers. This panel advised about the nature and comprehensibility of our questionnaire. Specifically, our questionnaire comprised three scales (i.e., institutional pressure, resource allocation, and legitimacy) and a variable measuring the dominant logic (i.e.,: high competitive or Sport-for-All); (d) the questionnaire was tested in a sample of ex-staff members of sport federations and club representatives. After the test phase, the questionnaire was addressed to the chief executive of each subsidized federation. In the end, 40 out of the 47 sport federations completed the questionnaire, representing a total response rate of 87.3%.

### Measurements

The questionnaire comprised three scales (i.e. institutional pressure, resource allocation, and legitimacy) and a variable indicating the dominant logic (i.e.,: high performance or Sport-for-All). These three scales and variable were constructed as set forth below:

#### Institutional pressure

A scale institutional pressure was constructed to measure in what fashion federations encounter pressure from their institutional context. To compose this variable four items were developed based on the theoretical overview. One example item was ‘since the enactment of the new decree on the sport federations our sport federation experiences more supervision from Sport Flanders on how we execute our sport policy’. This scale was shown to be a reliable instrument (Cronbach’s alpha = 0.658).

#### Dominant logic

In order to shed light in differences between the Sport-for-All and high performance logic, federations were asked to indicate the logic that best represent the main priority of their organization. The federations had three options. They had the possibility to answer that their organization was more competitive-oriented, Sport-for-All-oriented, or they could opt to select a remark field to answer why they did not agree with the first two options.

#### Resource allocation

This scale measured if the logic was indeed a priority in terms of resource allocation, such as budget, employees, infrastructure, and time investment. In particular, we measured the level of resource allocation using five items for high performance-oriented federations. An example item was*’*our sport federations spends the most of our budget on high performance’. This scale was shown to be a reliable instrument (Cronbach’s alpha = 0.636). For Sport-for-All-oriented federations, three items were created to measure resource allocation. An example items was ‘our sport federations spends the most of our budget on Sport-for-All’. This scale was shown to be a reliable instrument (Cronbach’s alpha = 0.738).

#### Legitimacy

Federations were asked if they get legitimacy from the institutional context for subscribing a specific logic. Five items were developed for federations with a competitive logic. An example items was ‘if our sport federation gets goods results on international tournaments we get recognition from other sport federations’. This scale was shown to be a reliable instrument (Cronbach’s alpha = 0.728). Three items were developed for federations with a Sport-for-All logic. An example of an item is: ‘Our sport federations is often asked for advice by other sport federations in how they should develop their Sport-for-All policies*’*. This scale was shown to be a reliable instrument (Cronbach’s alpha = 0.639.)

Separate principal components analyses (PCAs) were used to explore the factor structure of the institutional pressure, resource allocation, and legitimacy scales. These three scales each yielded one reliable factor. Only factor loadings higher than 0.4 were withheld in this study. Items with factor loadings lower than 0.4 were deleted from the analysis. Moreover, the PCAs and Cronbach’s alpha indicated that removing two of the five items within the resource allocation and legitimacy scale of the Sport-for-All federations would improve the internal consistency and factor structure of these scales, and consequently, the robustness and validity of our analyses. Therefore, only three items were used of the scale measuring resource allocation and legitimacy in Sport-for-All federations. The scales measuring resource allocation and legitimacy in high performance federations was not altered since these 5-item scales showed a satisfactory internal consistency and factor structure.

### Data analysis

Firstly, regarding the analysis of the consulted documents and websites, the policy documents and websites of sport federations were thematically analyzed to enhance our knowledge on the kinds of Sport-for-All projects the sport federations support [88]. To analyze the target groups of the Sport-for-All project, we opted to separate the target groups of the project. For example, when a project aimed to reach disabled and senior participants, we distinguished two separate target groups. Therefore, the number of target groups is higher than the number of unique Sport-for-All projects.

Secondly, to shed light on the tensions between the Sport-for-All and the high performance logic, we utilized the questionnaire addressed to the sport federations. Data analysis was conducted with SPSS Statistics 25. A multivariate analysis of co-variance (MANCOVA) was used to compare sport federations with a competitive logic and federations with a Sport-for-All logic. Institutional pressure, resource allocation, and legitimacy were included as the dependent variables. Organizational size (number of members) of the sport federations was added as a covariate.

## Results

### Sport-for-All projects

Based on the inclusion criteria, 218 Sport-for-All projects were distinguished by the 40 sport federations that conducted the survey, representing an average of 6.3 Sport-for-All projects per sport federation. The mapping also included Sport-for-All projects that were already supported for more than two decades such as start2run or start2tennis projects. The main goal of these ‘start2-projects’ was to allow participation free of cost in several training sessions to learn more about the sport and the sport club/ federation. The mapping also included more recent Sport-for-All projects. For example, the Gymnastics federation recently launched the freerunning project ‘as a way to attract sport participants who prefer light sport facilities and even disadvantaged communities. Because these groups still encounter a lot of barriers to participate in our clubs, we established freerunning communities as an intermediate step’. Being part of such communities entailed less regulatory and practical demands for the participants such as a fixed membership or being obliged to participate in the competitions formats of Gymfed.

### Target groups

The analysis showed that 58.5% of the projects addressed one specific target group, 11.8% addressed two target groups, and 29.6% of the projects were open for multiple target groups. The target group that was most addressed was *youth (under 18)* (29.1%), followed by *open for all* (26.3%) which refers to projects that are accessible for different kinds of target groups. Typical examples of such projects are the ‘*start2-projects*’, (e.g., Start2Run). Other popular target groups were *disabled participants* (11.6%) and *elderly* (10.4%). Less frequently addressed were disadvantaged communities such as *lower SES-groups* (4.8%) and *people with a migration background* (4.8%).

### Outcomes of the Sport-for-All projects

As mentioned by Coalter [26], Sport-for-All projects can pursue multiple outcomes. Our results demonstrated that all 218 Sport-for-All projects addressed the first two outcomes (i.e., remove of barriers to sport participation and opportunities to develop sporting skill). 28.9% of all projects provide a recreational competition, 28% of the projects included an educational program for the coaches, and 36.7% of the projects involved an external partnership.

### Multivariate MANCOVA-measurement

Concerning our second research question, 65% of the sport federations (e.g., soccer, athletics, and fencing) reported to subscribe a high performance logic, 27.5% of the sport federations (e.g., rugby, walking, climbing, and mountaineering) reported being oriented towards a Sport-for-All logic, and 7.5% sport federations explicitly self-reported having a holistic view on sport. As only 7.5% of the federations reported a holistic view, these federations were excluded from further analyses. Moreover, means and standard deviations among the scales are presented in Table 1.

**Table 1:** Descriptive statistics among variables

Scale	Variable	Mean	Std. deviation	N
**Institutional pressure**	Sport-for-All	9.45	2.544	11
	High performance	14.15	2.810	26
**Resource allocation**	Sport-for-All	11.45	2.067	11
	High performance	11.54	1.985	26
**Legitimacy**	Sport-for-All	13.00	4.405	11
	High performance	14.35	3.805	26

Furthermore, the MANCOVA-analysis revealed that the overall model was significant (Wilks’ Lambda = 0.59, F(7.369) = 0.00, *p* < 0.05). Moreover, the MANCOVA-analysis indicated a discrepancy in how federations with a high performance logic and those with a Sport-for-All logic responded to the current institutional pressure. In particular, the latter group endured more pressure than those with a Sport-for-All logic and this discrepancy was significant, F(23.077) = 0.00, *p* < 0.05. No significant difference was found for the scales resource allocation and legitimacy. Table 2 offers an overview of the MANCOVA-analysis.

**Table 2:** MANCOVA-analysis conducted on the sport federations

Multivariate test	Variable	*F*-value	Sig
	**-**	7.369	.001
**Univariate test**	**Institutional pressure**	23.077	.000
	**Resource allocation**	.033	.856
	**Legitimacy**	.776	.384

### Implications of the institutional logic on the Sport-for-All delivery

When combining the Sport-for-All projects with the underlying institutional logics of the sport federations, our analysis showed that the 26 sport federations with a high performance logic offer 66% of the Sport-for-All projects in total. The 11 sport federations with a Sport-for-All logic support 34% of the Sport-for-All projects. Moreover, these results were supplemented with the analysis of the strategic goals of the federations. This analysis revealed that both types were addressing Sport-for-All in their strategic target goals. The contrast lies in the fact that the high performance-oriented federations inserted more elite sport-oriented objectives in their strategic goals (e.g., ‘our federation wants to delegate at least one male or female at the Tokyo Olympic Games in 2020 via our performance program’). Furthermore, they referred less often to specific Sport-for-All projects in their strategic goals and when addressing Sport-for-All goals they were often formulated in general terms (e.g., ‘our federations will increase the number of recreational members by 100% by 2020, therefore we envisage a yearly increase of 25%’). This was in contrast with Sport-for-All-oriented federations who often addressed specific target groups (e.g., ‘by 2020 our federations wants to attain at least 50 members, of whom at least 20 refugees, with our climbing project’).

Furthermore, the specific target groups were linked to the underlying logic of the federations to indicate how the Sport-for-All and high performance-oriented federations aimed to reach specific target groups. The accompanying results are summarized in Table 3.

**Table 3:** Target groups aimed at by the Sport-for-All projects

Target group	Sport federations with a high performance logic		Sport federations with a Sport-for-All logic	
	**% of representation in the projects**	**Mean of projects per sport federation**	**% of representation in the projects**	**Mean of projects per sport federation**
**Open for all**	28.7	2	22.9	1.8
**Youth**	30.9	2.1	16.7	1.3
**Seniors**	6.2	.4	20.8	1.7
**Disabled**	11.8	.8	9.4	.8
**Adults**	6.2	.4	3.1	.3
**Coaches**	3.4	.2	3.1	.3
**Females**	2.8	.2	2.1	.3
**Families**	2	.1	5.2	.4
**participants with a migration background**	3.4	.2	7.3	.6
**Companies**	1.7	.1	1.0	.1
**Students**	.6	.0	.0	.0
**Lower SES-groups**	2.8	.2	8.3	.7

The results presented in Table 3 are also reflected in the analysis of the strategic target goals. When high performance-oriented federations mentioned specific target groups in their strategic goals, they mostly referred to youth. Sport-for-All-oriented federations more often addressed disadvantaged groups, such as lower SES-groups, seniors, and participants with a migration background in their strategic objectives.

Finally, the specific outcomes of the projects were linked to the subscribed logic. Sport federations with a high performance logic provided 28.4% of their projects in a recreational competition. Moreover, an extra supportive program for the coaches was available in 23.9% of their projects, while 30.3% of their projects relied on assistance of an external partner. In the case of Sport-for-All-oriented federations, 21.5% of their projects included a recreational competition, 24.1% of their projects foresaw in extra education for the coaches, and they relied on an external partnership in 35.4% of the projects.

## Discussion

Our results illustrated, as an answer to our first research question, that the Flemish sport federations deliver major contributions to the Sport-for-All delivery in quantitative terms. However, in qualitative terms, the federations and their projects often lack the intention to include specific disadvantaged groups, such as participants with a migration background and lower SES-groups. This is especially the case in high performance-oriented federations. In particular, our study aimed to strengthen understanding of which implications the underlying institutional logic, being a high performance or Sport-for-All logic has on these federations Sport-for-All projects. As such, one of the main results of this study is that most federations apply a high performance logic. The fact that the underlying logic of most federations is high performance-oriented, may be an explanation why federations struggle to attain their Sport-for-All goals. This reasoning can be linked with the thoughts of several leading authors in the field of Sport-for-All, such as Spaaij [89], Skille [14, 90], Stenling and Fahlén [83]. These authors are critical to a so-called ‘double track approach’ that is expressed in several national sport policies. This ‘double track approach’ refers to the complex issue of balancing the implementation of Sport-for-All with the stimulation of elite sport achievements in the same organization. The abovementioned authors emphasize that the Sport-for-All ideal is hard to realize in those organizations, because the logic of high performance comprises various processes of elitism, selection, and exclusion [14, 83, 89–91]. Based on their thoughts, we assumed that sport federations that subscribe to a high performance logic would not or only barely contribute to the Sport-for-All delivery. However, our results demonstrate that the gap between high performance and Sport-for-All-oriented federations is smaller than expected for several reasons.

Firstly, the engagement of high performance-oriented sport federations in the Sport-for-All delivery may be seen as a remnant of their past commitment in the early Sport-for-All campaigns, and of their obligation to execute governmental policies related to the Sport-for-All [5]. Flemish sport federations still get general subsidies in exchange for fulfilling basic tasks, as laid down in the decree that prescribes their operation [5]. One of these basic tasks is that sport federations have to offer Sport-for-All. Furthermore, this dual task of high performance-oriented federations is also embodied in their strategic goals. In contrast to the Sport-for-All-oriented federations—which are to a lesser extent, or (in some cases) not addressing competitive related target goals—the high performance-oriented federations adopt a staggered position when it comes to the tension between Sport-for-All and competitive target goals. Moreover, this accords with the results of the MANCOVA-analysis. The analysis indicated that high performance-oriented federations currently respond significantly more to institutional pressure than Sport-for-All-oriented federations. An explanation for this result may relate to the fact that high performance-oriented federations are obliged to assist in the Sport-for-All delivery, while they aim to be successful on the international elite sport scene as well. The existing scholarship on institutional theory points out that organizations can manage multiple (and at times) competing institutional logics [72, 80, 92]. Furthermore, Kraatz and Block (2008) stated that organizations (outside the sport context) often endorse different institutional logics to conform to the varying amount of pressures they experience from the institutional context [93].

Secondly, when closely analyzing the specific target groups of the Sport-for-All projects, our study offers an intriguing result. More specifically, that most projects of the high performance-oriented sport federations are focused on youth, open for all, and disabled sport participants. These target groups are – perhaps unsurprisingly—closely linked to their competitive core business [94–96]. It might be possible that federations with a competitive logic organize Sport-for-All projects, while applying the projects as a detection system for (future) sport talents. This hypothesis corresponds with research conducted on elite sport development, showing that a lot of elite athletes have their roots in Sport-for-All projects and Sport-for-All is often applied as a trickle-up mechanism to generate elite athletes [96, 97].

Conversely, Sport-for-All-oriented federations deliver more specific projects to include the target groups that are often excluded from organized sport participation. Their efforts are much more in accordance with how disadvantaged groups are represented in our society. For instance, more than eight percent of the inhabitants of Flanders do not possess the Belgian nationality [98].

Thirdly, although the high performance-oriented federations are providing Sport-for-All projects, our study identified a potential discrepancy in terms of quality compared to projects of federations with a Sport-for-All logic. Our analysis of the outcomes exposed that competitive-oriented federations more often implement a competitive element in their projects, whereas Sport-for-All federations focus more on educational programs for the coaches and on external partnerships. With regard to disadvantaged groups, international literature acknowledges that these target groups often reject a competitive setting, because it comprises components similar to those that they have already rejected [99, 100]. Moreover, high performance-oriented federations may not be the most preferable settings to include these target groups. Furthermore, research revealed that the attitude and experiences of the coach play an important role in reaching these specific groups. Therefore, it is possible that the additional education programs for coaches in Sport-for-All federations are more oriented towards the young people’s well-being and their specific needs and life situations, while the education programs in high performance-oriented federations are more fixated on competition [101].

Although most sport federations developed their Sport-for-All policies since the 1970s, the goal of Sport-for-All has still not been fully accomplished [89]. This observation raises questions on the future role of sport federations in the Sport-for-All delivery. On the one hand, sport federations have to deal with a heterogeneous amount of tasks such as offering high performance sport opportunities, professionalizing their sport clubs because these clubs are mostly managed by volunteers, finding new ways to make their sport more attractive, and looking for funding or financial support [13, 102]. Consequently, the heterogeneity and complexity of these tasks may hinder a more in-depth elaboration of their Sport-for-All policies. Especially, targeting disadvantaged communities is time consuming and intensive. Hence, federations that want to successfully include these target groups will have to clearly define their organizational priorities. On the other hand, federations have incorporated a lot of experience on how to guide and coach sport participants. Moreover, policy makers should reassess how they envisage the role of sport federations and especially the role of high performance-oriented federations in the Sport-for-All delivery. Because the latter federations have to contribute to a dual mission these federations are characterized by more superficial Sport-for-All projects compared to the Sport-for-All oriented federations. If policy makers want these federations to elaborate their Sport-for-All policies to attain social and public health targets, our suggestion is to separate logics into different organizations or divisions, which creates space for the (sub)organizations to solely focus on their dominant logic. This can have multiple advantages. High performance divisions can exclusively focus on improving the quality of their competitions and elite athletes programs, whereas Sport-for-All-oriented divisions can aim more fully on reaching disadvantaged groups. In support of our suggestion, we would like to point to the example of Denmark. In particular, Denmark is characterised as a country with a relatively high level of sport participation. Denmark differs from other sport systems in Europe due to a stronger separation between the organizations that focus on elite sport and the organizations that support Sport-for-All [103]. In the Danish sport system, two organizations focus on elite sport. First, there is ‘Team Denmark ‘ which solely promotes and supports elite sport. Second, there is the National Olympic Committee and Sport Confederation of Denmark (DIF). The DIF comprises the national sport federations that have most of their interest and money go to elite sport. For the development of Sport-for-All, the Danish system relies on the Danish Gymnastics and Sports Associations (DGI), which is the umbrella organisation for 15 regional associations that focus solely on offering Sport-for-All [103, 104]. However, this does not implicate that performance-oriented federations should be left completely out of the Sport-for-All delivery. These federations have incorporated several capacities such as well-elaborated talent recruitment and development systems, the availability of accommodation, and knowledge on how to train sport participants. In addition, performance-oriented federations are responsible for organizing the sport competitions of popular sports such as gymnastics, soccer, and athletics. Consequently, leaving these federations totally out of the Sport-for-All delivery would be a missed opportunity. Moreover, policymakers should develop strategies about how both types of federations could strengthen each other, for example by stimulating cooperation between them. This is especially important for sport participants who do not (longer) flourish in a competitive setting. Then performance-oriented federations should advise and guide these participants to a Sport-for-All-oriented federation. This way, a win–win solution can be created for both types of federations and the Sport-for-All landscape in general.

## Conclusions

By exploring the role of sport federations contributions in the Sport-for-All delivery, our study helps to expand the literature in a twofold way.

Firstly, this study provides insights in the amount of Sport-for-All projects the Flemish sport federations support and organize. Moreover, the conducted mapping of projects offers findings on which specific target groups the projects aim to reach. Our study indicates that the high performance federations especially target groups like youth or develop provision that is open for all. Their Sport-for-All projects lack a focus on disadvantaged groups, whereas these groups are more represented within the project of Sport-for-All oriented-federations.

Secondly, previous research emphasized the importance of more theoretically guided and empirical research to provide a better understanding of different sport-related logics and their implications [14]. This paper attempts to contribute to this call. We developed a questionnaire to measure institutional theory in a quantitative way in federations. Furthermore, by providing knowledge on the impact of the underlying logic on federations Sport-for-All projects and revealing that high performance-oriented federations show a significantly higher response to current institutional pressures, our results render a theoretical implication of institutional theory on sport federations.

### Limitations and further research

Although this study rendered interesting conclusions, we need to address some limitations as well. Firstly, we focused on the tension between the high performance and Sport-for-All logic in the context of sport federations. We must emphasize that other types of institutional logics can also operate in the institutional context of sport federations such as a commercial or governmental logic [13]. We chose to discard these other institutional logics to not diffuse the main focus of our research which is investigating the tensions between high performance and Sport-for-All logics.

Secondly, the paper provides insights in the Sport-for-All delivery at a given time, but results of the same study at a different time might differ, and therefore multiple points of measurement over a period of time would provide us with the means to analyze if sport federations’ contribution to the Sport-for-All delivery is declining or increasing and if they succeed to reach more disadvantaged groups in the future.

Thirdly, because a clear definition of a Sport-for-All project is lacking, we applied broad criteria to select the Sport-for-All projects. This is also the case for the Coalter outcomes applied in this study. This non-definitive list was distinguished by Coalter [26] based on analyzing sport programs that aimed to improve the sporting inclusion and are therefore not specifically oriented towards Sport-for-All outcomes. Furthermore, we only applied the outcomes of Coalter in our study which can be considered a limitation. Fourthly, because the mapping was based on a questionnaire, policy documents, annual reports, and websites of the federations, it is hard to draw conclusions about the execution and scale of the Sport-for-All projects in practice. Lastly, we analyzed the Sport-for-All policies of federations based on their projects but this does not implicate that federations, apart from their projects, may not deliver excellent work in the broader Sport-for-All area. This last limitation leads to recommendations for further research. We raise the call to conduct more research that provides knowledge on whether there is a difference in terms of quality and scale between the project of federations with a Sport-for-All logic and federations with a high performance logic.

A second recommendation is that our criteria to select the Sport-for-All projects were broad so future studies could elaborate on the criteria used in this study. Furthermore, Coalter’s list of outcomes is not an exhaustive list so future studies could supplement and help to identify (new) outcomes of Sport-for-All projects. Moreover, more research can be conducted on institutional change in sport federations e.g. how can a high performance-oriented federations make the shift towards a Sport-for-All-oriented federation? Or, elaborating on our suggestion of separating the competing logics in different organizations, more research can be conducted on how this change process has to evolve within sport federations. Furthermore, a longitudinal research design could provide more knowledge on how Sport-for-All projects evolve over time.

## Data Availability

The dataset analysed in the current study is not publicly available, or available on reasonable request from the corresponding author because participants explicitly consented to only have their data shared with the immediate research team.
